# The Protective Effects of Shen-Fu Injection on Experimental Acute Pancreatitis in a Rat Model

**DOI:** 10.1155/2014/248786

**Published:** 2014-03-05

**Authors:** Lei Huang, Jun Cao

**Affiliations:** ^1^Department of Plastic Surgery, Zhongnan Hospital of Wuhan University, 169 Donghu Road, Wuchang, Wuhan, Hubei 430071, China; ^2^Department of General Surgery, Zhongnan Hospital of Wuhan University, 169 Donghu Road, Wuchang, Wuhan, Hubei 430071, China

## Abstract

*Objectives*. In the present study, we investigated the protective effects of Shen-Fu injection (SFI) on a caerulein-induced rat pancreatitis (AP) model. *Methods*. SFI was given to rats in the SFI treated group through intraperitoneal injection. Blood and pancreas samples were collected for serological and histopathological studies. *Results*. Our results showed that AP caused significant decrease in tissue glutathione (GSH) and serum IL-4 and IL-10, while pancreatic malondialdehyde (MDA) and myeloperoxidase (MPO) were increased. Furthermore, TNF-**α**, IL-1**β**, amylase, and lipase levels were also significantly increased. On the other hand, SFI treatment reserved all these biochemical indices as well as histopathologic alterations that were induced by caerulein. *Conclusion*. Our findings suggest that the SFI protects against caerulein-induced AP in rats via modulation of cytokines, oxidative stress, and Nuclear Factor-kappa B (NF-**κ**B) activity.

## 1. Introduction

Acute pancreatitis (AP) involves a complex cascade of events and the pathogenesis of AP remains still obscure and multifactorial. To date, most investigators believe that AP is caused by the unregulated activation of trypsin within pancreatic acinar cells, and enzyme activation within the pancreas leads to the autodigestion and local inflammation [1]. The proinflammatory cytokines (such as IL-1*β* and TNF-*α*) and the anti-inflammatory cytokines (such as IL-4 and IL-10) have been shown to be intimately involved in the inflammatory response to AP [2]. Moreover, NF-*κ*B is activated during early stages of pancreatitis and regulates many genes that may control inflammatory activities. Most recently, a study showed that the level of NF-*κ*B activation correlates with the severity of AP [3]. Although there is no specific treatment available for AP, numerous pharmacologic therapies have been evaluated over the past few decades. A few of them such as anti-inflammatory medications and antioxidants have demonstrated promise in significantly altering the progression of AP [4], and the search for specific therapies continues.

Shen-fu injection (SFI) is an extract of traditional Chinese herbs, which mainly consists of ginsenoside and aconitine. In China, SFI is applied routinely in clinical practice in treating coronary heart disease and congestive heart failure (CHF) [5]. Several studies showed that SFI has protective effects against Ischemia-reperfusion injury (IRI) on many organs such as heart, liver, kidney, and brain [6–9]. Wang et al. reported that SFI might reduce the expressions of TNF-*α* and IL-6 in rats with systemic inflammatory response syndrome (SIRS) via inhibiting the activity of NF-*κ*B [10]. A very recent study has also proven that SFI could suppress inflammation in rats with CHF [11].

Therefore, we hypothesize that SFI has protective effects on AP, partly by mediating anti-inflammatory actions. To test this, we used a caerulein-induced rat AP model and investigated the expression of cytokines and the activity of NF-*κ*B. We also measured the serum amylase and lipase activity, myeloperoxidase activity, malondialdehyde expression, and histological damage.

## 2. Materials and Methods

### 2.1. Ethics Statement

This study was performed in strict accordance with the recommendations in the Guide for the Care and Use of Laboratory Animals of Wuhan University. The protocol was approved by the Animal Experiments Ethics Committee of Wuhan University (permit number 2012030). All of the surgeries were performed under sodium pentobarbital anesthesia, and all efforts were made to minimize suffering.

### 2.2. Animal Model and Study Design

Thirty male specific pathogen-free (SPF) Sprague Dawley (SD) rats weighing 180~200 g were obtained from the Animal Biosafety Level III laboratory (ABSL-III lab) of Wuhan University in China. Rats were housed individually in cages on a 12 h dark/12 h light cycle at 23 ± 2°C under standard environmental conditions and had free access to pellet diet and tap water. Rats were randomly divided into control group (*n* = 10), caerulein group (*n* = 10), and SFI group (*n* = 10 in each subgroup). As previously described [12], four subcutaneous injections of caerulein (Sigma-Aldrich, MO, USA; 20 *μ*g kg^−1^ body weight) or vehicles (normal saline) were applied consecutively at 2 h intervals. Shen-Fu Injection (SFI) was produced by Ya'an Sanjiu Pharmaceutical Co., Ltd. (Ya'an, China). The main components of Shen-Fu injection include ginsenoside (>0.8 mg/mL) and aconitine (<0.1 mg/mL). SFI (1 mL, 5 mL or 10 mL/kg body weight in SFI subgroup 1–3, resp.) or equivalent normal saline (10 mL/kg body weight in caerulein) was given to rats through once intraperitoneal injection at the time of the last injection of caerulein. To ensure that rats receive equivalent liquid volume in all groups, Shen-Fu injections used in the SFI subgroup1 and subgroup 2 were diluted with normal saline into 10 mL/kg body weight. Rats were sacrificed with an overdose of pentobarbital 24 h after the last injection of caerulein for blood and pancreas samples collection.

### 2.3. Measurement of Amylase, Lipase, and Cytokines in Serum

Blood samples were centrifuged at 15,000 rpm under 4°C and then stored in a –80°C fridge. Serum amylase and lipase were measured with a TBA-2000FR System (Toshiba, Tokyo, Japan). The serum cytokines (TNF-*α*, IL-1*β*, IL-4, and IL-10) were detected using a commercial enzyme-linked immunoabsorbent assay (ELISA) kits (Boster, Wuhan, China), according to the manufacturers' protocols.

### 2.4. Pancreatic Myeloperoxidase, Malondialdehyde, and Glutathione

The pancreatic myeloperoxidase (MPO) activity was determined as described [13]. The resulting change in absorbance at 460 nm was measured spectrophotometrically for 5 min. One unit of MPO activity was defined as that degrading 1 mmol of peroxide per min at 25°C. The activity was expressed as U/mg of tissue. The malondialdehyde (MDA) was determined using the thiobarbituric acid test [14]. The absorbance was measured at 532 nm. The results were expressed as nmol/g of tissue. The GSH content was measured using the 5,5′-Dithiobis(2-nitrobenzoic acid)-oxidized GSH (DTNB-GSSG) reductase recycling assay for total glutathione (GSH+GSSG) as described by Tietze [15].

### 2.5. Western-Blot Assay for NF-*κ*B p65

Total proteins were purchased from pancreatic tissue using a total protein extraction kit (Boster, Wuhan, China). Protein concentrations were determined using a commercial kit (Pierce, Rockford, IL, USA). Each 20-*μ*g aliquot of total protein was loaded onto sodium dodecyl sulfate-polyacrylamide gel for electrophoresis and then transferred onto membranes. Following complete protein transfer, the membranes were blocked with 5% milk powder solution for 2 h and incubated with NF-*κ*B p65 primary antibody (Santa Cruz Biotechnology, Inc., Dallas, TX, USA) overnight. After washing the membranes, the secondary antibody (Boster, Wuhan, China) was applied and incubated for 2 h at room temperature (RT). Bands were quantified by a calibrated imaging densitometer (GS-710; Bio-Rad) and analyzed by “Quantity One” software (Bio-Rad). Glyceraldehyde-3-phosphate dehydrogenase (GAPDH) was used as an internal reference.

### 2.6. Evaluation of Histological Damage

Pancreas tissues were collected for morphological evaluation. The specimens were fixed in 10% formalin, embedded in paraffin, cut into sections 4-microns in thickness, and stained with hematoxylin and eosin (H&E). Five observation fields under light microscope were randomly selected in each specimen and were blindly evaluated by two pathologists after randomization. All specimens were photographed using a digital camera (450D, Canon, Tokyo, Japan) and scored through the method of Schmidt et al., 1992 [16]. Briefly, the morphological injuries to pancreas were evaluated through five main aspects: edema, acinar necrosis, inflammatory cell infiltration, hemorrhage, fat necrosis, and perivascular inflammation. Scores range from 0 to 4 for each parameter, and high scores indicate severe damages. Final scores were calculated and used for statistical analysis.

### 2.7. Statistical Analysis

Data are expressed as mean ± SD. The data was processed by the statistical analysis software SPSS version 16.0 (SPSS Inc., Chicago, IL, USA). Comparison of several mean values was performed using one-way and repeated measure two-way analysis of variance followed by the Tukey-Kramer test to identify significant difference between groups. All *P* values were two-tailed and a *P* value of less than 0.05 was considered significant.

## 3. Results

### 3.1. Serum Amylase and Lipase

**Figure 1:**
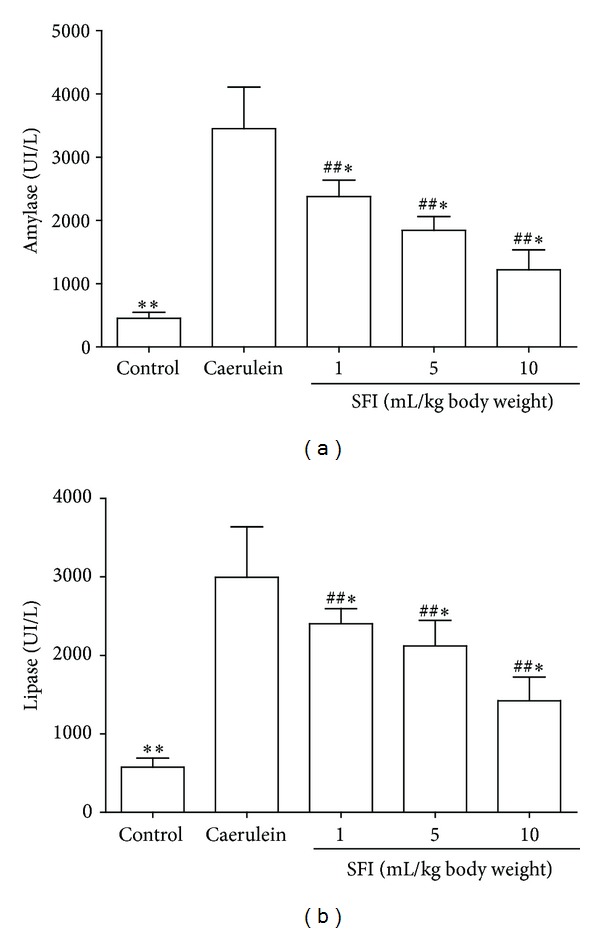
Serum expressions of amylase and lipase. (a) Serum amylase expression. (b) Serum lipase expression. Both serum amylase and lipase activities were significantly increased in the caerulein group, while treatments with SFI cause those enzymes to revert to basal levels. Compared with the caerulein group, ^#^ *P* < 0.05, ^##^ *P* < 0.01; compared with other groups, *P < 0.05, **P < 0.01.

The serum levels of amylase and lipase are shown in Figure 1. Compared to the control group, amylase and lipase significantly increased in caerulein groups (*P* = 0.001). Treatment with SFI causes those enzymes to revert to basal levels. Compared to the caerulein group, both amylase and lipase decreased in the SFI treated groups (*P* < 0.01, resp.). In addition, serum amylase and lipase decreased with increasing dosages of SFI in SFI treated groups.

### 3.2. Serum Cytokines

**Figure 2:**
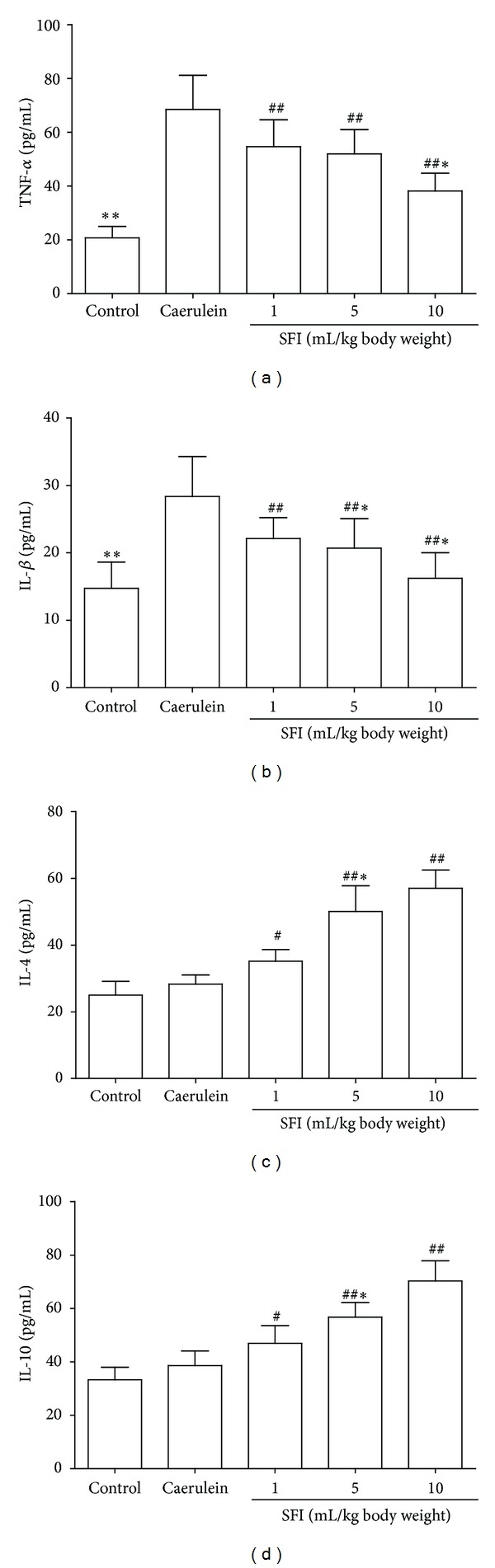
Serum concentrations of cytokines. (a) Serum concentrations of TNF-*α*. (b) Serum concentrations of IL-1*β*. (c). Serum concentrations of IL-4. (d) Serum concentrations of IL-10. Compared to the control group, the proinflammatory cytokines TNF-*α* and IL-1*β* were significantly increased, while the anti-inflammatory cytokines were insignificantly increased in the caerulein group. Treatments with SFI inhibited the expression of proinflammatory cytokines TNF-*α* and IL-1*β* and improved the expression of anti-inflammatory cytokines IL-4 and IL-10. Compared with the caerulein group, ^#^ *P* < 0.05, ^##^ *P* < 0.01; compared with other groups, *P < 0.05, **P < 0.01.

Caerulein significantly elevated the serum proinflammatory cytokines (TNF-*α* and IL-1*β*), whereas the serum anti-inflammatory cytokines (IL-4 and IL-10) were insignificantly increased (*P* < 0.01, resp.). SFI significantly inhibited the secretion of proinflammatory cytokines IL-1*β* and TNF-*α* and improved the serum anti-inflammatory cytokines IL-4 and IL-10 (*P* < 0.05 and *P* < 0.01, resp.) (Figure 2).

### 3.3. Pancreatic Myeloperoxidase Activity, Malondialdehyde, and Glutathione Expressions

**Figure 3:**
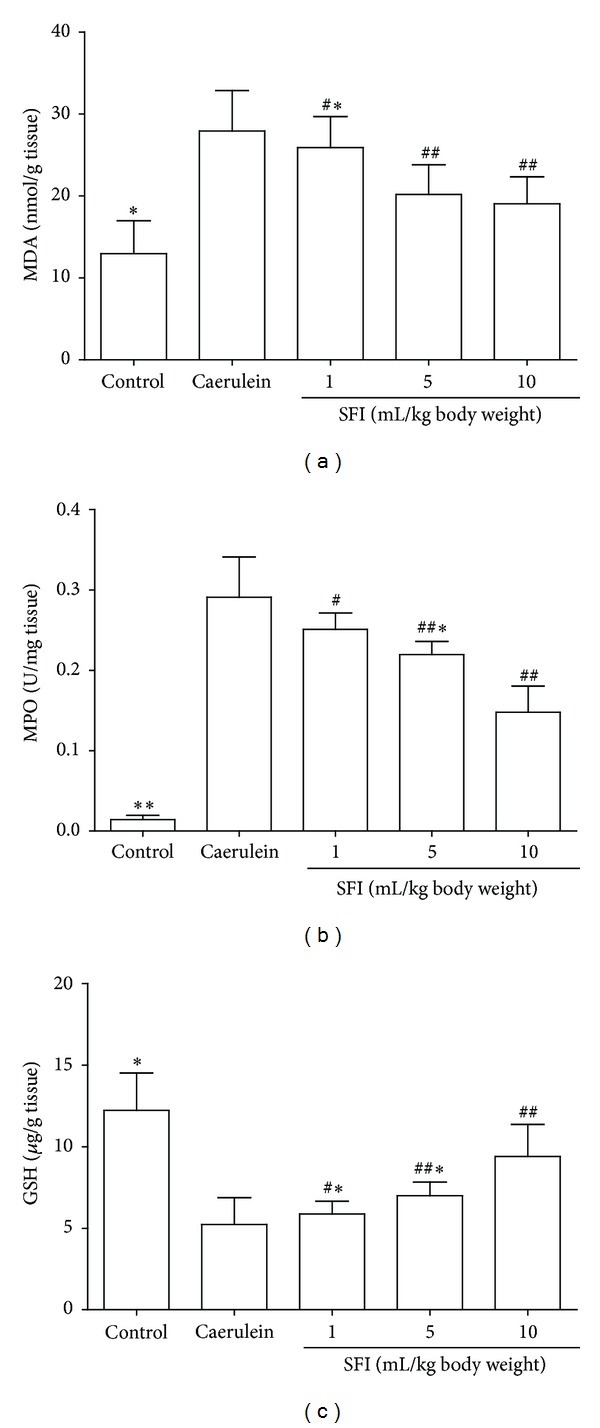
Pancreatic myeloperoxidase, malondialdehyde, and glutathione. (a) Pancreatic myeloperoxidase (MPO) activity. (b) Pancreatic malondialdehyde (MDA) expression. (c) Pancreatic glutathione (GSH) expression. Treatments with SFI reduced the MPO activity and MDA expression in pancreas. In addition, the expression of GSH was significantly increased in SFI treated groups. Compared with the caerulein group, ^#^ *P* < 0.05, ^##^ *P* < 0.01; compared with other groups, *P < 0.05, **P < 0.01.

Caerulein caused a significant increase in pancreatic MPO activity and MDA expression and a decrease of pancreatic glutathione (GSH) concentrations (*P* < 0.01, resp.). However, these changes were reversed by the treatment with SFI in a significant dose-dependent manner (*P* < 0.05, resp.) (Figure 3).

### 3.4. NF-*κ*B p65 Expression in Pancreatic Tissues

**Figure 4:**
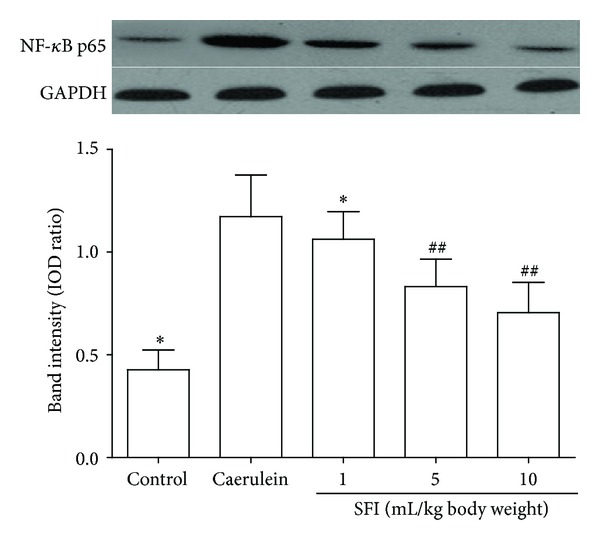
Expression of NF-*κ*B p65 in pancreas. Compared with the control group; the expression of NF-*κ*B p65 was significantly increased in the caerulein group. The treatments with SFI significantly reduced the expression of NF-*κ*B p65 in the SFI treated groups. Compared with the caerulein group, ^##^ *P* < 0.01; compared with other groups, *P < 0.01.

Compared to the control group, the expression of NF-*κ*B p65 in the caerulein group was significantly higher (*P* < 0.01). However, the expression of NF-*κ*B p65 has been inhibited in SFI treated groups. Compared to the caerulein group, NF-*κ*B p65 expression in the SFI treated groups was significantly lower (*P* < 0.01, resp.). In addition, the expression of NF-*κ*B p65 significantly decreased with increasing dosages of SFI (Figure 4).

### 3.5. Evaluation of Histological Damage

**Figure 5:**
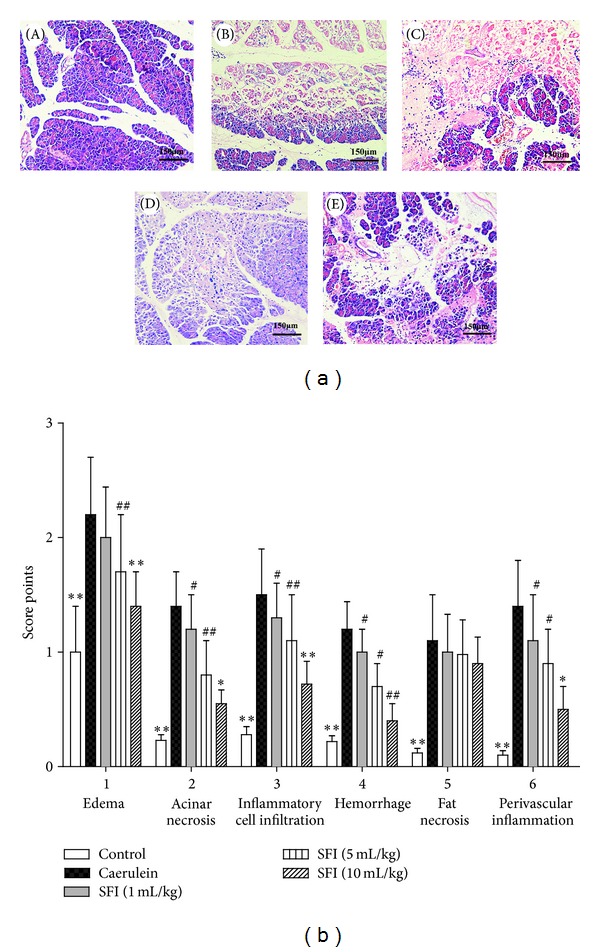
Histological evaluation. (a) Light microscope observations of H&E stained sections (×200). (A) Control group: normal pancreatic structure was observed. (B) Caerulein group: severe edema, inflammation cell infiltration, and acinar necrosis were noted. (C), (D), and (E) SFI subgroup 1–3, respectively: SFI treatments reduced pancreatic edema, inflammation and acinar necrosis. (b) Histological scores. Morphological injuries to pancreas were evaluated through five main aspects: edema, acinar necrosis, inflammatory cell infiltration, hemorrhage, fat necrosis, and perivascular inflammation. SFI treated groups showed significantly lower scores of those parameters except the fat necrosis. Compared with caerulein group, *P < 0.05, **P < 0.01; compared with control group, ^#^ *P* < 0.05, ^##^ *P* < 0.01.

Light microscope observations of H&E stained sections revealed the presence of edema, acinar necrosis, hemorrhage, fat necrosis, inflammation, and perivascular infiltrate. The normal control showed normal architecture and absence of edema, leukocyte infiltration, acinar vacuolization, hemorrhage, and necrosis. In contrast, pancreatic sections from the caerulein group revealed extensive tissue damage and thus received significantly higher histologic scores (*P* < 0.01, resp.). The treatment with SFI reduced the inflammation and edema. In addition, SFI treated groups showed significantly lower pathological scores (*P* < 0.05, resp.) (Figure 5).

## 4. Discussion

In this study, we used a caerulein-induced rat acute pancreatitis model to investigate the protective effects of SFI. Our results demonstrated that SFI inhibited the production of proinflammatory cytokines, the expression of NF-*κ*B, and serum levels of lipase and amylase in caerulein-induced rat acute pancreatitis. The treatment with SFI also caused the serum anti-inflammatory cytokines, pancreatic MPO activities, and MDA concentrations to significantly revert. In addition, rats which received SFI treatment showed improved pancreatic morphology.

AP is an inflammatory disease with wide clinical variations, which may present sepsis, multiple organ failure, and even death [17]. During AP, amylase and lipase are released from acinar cells, and their concentration in the serum is used to confirm diagnosis [18]. In this study, the serum amylase and lipase in the caerulein-treated rats exceeded three times the normal upper limit. In addition, very high MPO activity and MDA expression with elevated GSH concentrations were also observed in the caerulein-induced rats, indicating that caerulein induces an oxidative stress. These results support that the AP model has been successfully established in our study. Importantly, our study showed that SFI was able to cause serum lipase and amylase to revert to basal levels, which suggests that SFI has the potential to inhibit pancreatic injury.

Oxidative stress is involved in the pathogenesis of AP [19]. Previous studies have shown that oxygen free radicals (OFR)-induced lipid peroxidation and changes in GSH metabolism occur at an early stage in the course of AP [20, 21]. Antioxidant therapies have been shown to improve pancreatitis induced by caerulein administration [22]. GSH is the main nonprotein thiol within the cell, part of a major intracellular defense system against oxygen free radicals (OFR) [23], and the enhanced expressions of MDA as a result of membrane lipid peroxidation may indirectly reflect OFR activity [24]. In our study, the observed decreases in the MPO activity and MDA expression with increased GSH concentrations in SFI treated rats suggests that SFI protects the pancreatic tissues from oxidative damage induced by caerulein, and this effect possibly involves the inhibition of neutrophil infiltration and lipid peroxidation.

Previous studies are mainly focused on the protective effects of SFI against I/R injuries. Recent studies also show that SFI has potential anti-inflammatory ability [10, 11]. Proinflammatory cytokines, such as IL-1*β* and TNF-*α*, play a pivotal role in the early pathophysiological events of AP. Those cytokines initiate and propagate almost all consequences of the SIRS [25]. This study and other previous reports have detected the increased serum levels of IL-1*β* and TNF-*α* in both human and laboratory animal AP [26, 27]. In the current study, the serum proinflammatory cytokines IL-1*β* and TNF-*α* were significantly decreased due to the treatment with SFI. In contrast, the anti-inflammatory cytokines IL-4 and IL-10 were significantly increased in SFI treated groups. Those results suggest that SFI plays an anti-inflammatory role in AP. NF-*κ*B is a nuclear transcription factor responsible for regulating the transcription of a wide variety of genes involved in immunity and inflammation. It has been reported that NF-*κ*B activity in pancreatic acinar cells plays a role in the inflammatory response that occurs during acute pancreatitis [28] and selective NF-*κ*B inhibition resulted in decreased inflammation in the pancreas [29]. Our previous study also showed that inhibition of NF-*κ*B in AP with sivelestat resulted in a better outcome for rats [27]. Additionally, SFI may reduce the levels of inflammatory mediators by inhibiting NF-*κ*B [10]. In our study, NF-*κ*B expression in pancreas has been inhibited by the treatment with SFI. Thus, the anti-inflammatory effect of SFI is possibly associated with its ability to inhibit the expression of NF-*κ*B.

## 5. Conclusion

In this study, SFI showed a dose-dependent protective effects in rat acute pancreatitis, which may exert an anti-inflammatory effect and reduce the histological damage due to its ability to regulate the production of cytokines, the oxidative stress, and the NF-*κ*B activity and to cause MPO, MDA, and GSH to revert to control levels. In addition, the anti-inflammatory ability of SFI in acute pancreatitis might be related to the inhibition of NF-*κ*B. Thus, SFI may have a protective effect against acute pancreatitis via modulation of cytokines, oxidative stress, and NF-*κ*B activity.
